# Exploring patients’ pharmacy stories: an analysis of online feedback

**DOI:** 10.1007/s11096-021-01287-2

**Published:** 2021-06-19

**Authors:** Jared Loo, Georgina Greaves, Penny J. Lewis

**Affiliations:** Division of Pharmacy and Optometry, Faculty of Biology, Medicine and Health, Manchester Academic Health Science Centre, School of Health Sciences, University of Manchester, Stopford Building Oxford Rd, Manchester, M13 9PT England UK

**Keywords:** Online patient feedback, Patient experience, Qualitative research

## Abstract

*Background* Studies have demonstrated the potential for patient feedback to inform quality care as well as a direct relationship between patient experience and clinical outcomes. Over recent years, there has been increasing use of online patient feedback platforms, however, there has been little study of the content of patient feedback relating to pharmacy and pharmacy services. *Objective* This study explores the content of online feedback provided by patients from across the UK in relation to their experiences of their interaction with pharmacy staff and pharmacy services. *Main outcome measure* Content of online patient feedback relating to pharmacy. *Method* Patient stories published on Care Opinion, a national online patient feedback platform, for a one-year period were searched for all content relating to patients’ pharmacy experiences. A thematic and sentiment analysis was conducted on 237 patient stories. *Results* Patient stories related to supply, staff attitudes, services, accessibility, systems, and errors. Patient sentiment depended on pharmacy setting, but staff attitudes, services, and accessibility were generally positive across all settings. Waiting time was the most common complaint in both hospital and community pharmacies with stories relaying experiences of slow discharge, stock shortages and poor communication and collaboration between pharmacies and GP surgeries. *Conclusions* Online patient feedback highlighted factors important to patients when interacting with pharmacies and their staff. Medication supply was the primary topic of patient stories with waiting times and stock shortages being clear areas for improvement; however, accessibility, pharmacy services and advice were key strengths of the profession. Further research is needed to understand how online patient feedback can be used effectively to inform improvements in pharmacy services.

## Impact of findings

- Patients’ online stories could be helpful to pharmacy organisations demonstrating what patients’ value in their interactions with pharmacy services and staff as well as the problems that they encounter.
- Improved communication between GPs and community pharmacies as well as more effective systems to support efficient discharge from hospital are areas that require attention in order to improve patient experiences.
- Pharmacy organisations should consider how to best engage with, and make use of, online patient feedback in order to drive improvements in patient care.

## Introduction

Over the last two decades, healthcare policy in the UK has emphasised the importance of capturing patient experience and feedback in order to drive quality improvement [1]. The use of inpatient surveys across all acute hospitals in England [1, 2] has been widespread since 2002. Patient feedback is also gathered across community pharmacies in England via the community pharmacy patient questionnaire (CPPQ). The CPPQ forms part of the conditions of England’s Community Pharmacy Contractual Framework (CPCF), the results of which should inform pharmacy contractors on how to improve their services.

With the digital revolution underway, new ways of gathering patient experience data online have been promoted alongside the traditional use of paper surveys [3, 4]. This shift in the landscape of patient experience has seen the increasing use of patient feedback websites [5–7]. Websites such as Care Opinion and the National Health Service (NHS) website are now widely used by patients and providers as a platform for providing feedback on healthcare services. Such websites allow patients to share their insights into care at a time and place convenient for them and in a manner that allows for guaranteed anonymity. This digitalisation may also boost transparency and facilitate sharing of data between patients and healthcare providers, allowing patients to be more responsible and autonomous in managing their health as well as providers improving safety and quality of care [5] in line with the UK’s NHS Long Term Plan [8].

Healthcare professionals, particularly general practitioners (GPs), have expressed their concerns that feedback is unreflective of the true performance of an organisation and the potential for negative user-driven content to cause reputational damage [4, 7]. Hence, the majority of GPs rarely encourage patients to leave online feedback due to fear of it becoming an outlet for disgruntled patients [10], despite studies reporting that a patient feedback is often positive [4, 9–13]. Some healthcare staff do meaningfully engage and respond to patient feedback [14], however, there is little understanding of engagement with patient feedback in the pharmacy sector.

To date, there have been relatively few studies of the content of online patient feedback, and none undertaken in relation to patient feedback relating to pharmacy. The collation and analysis of patient feedback data from across the UK is a useful exercise, facilitating understanding of the broad issues experienced by patients when interacting with pharmacy services, with potential for shaping future development and improvement within the sector. Previous research tends to focus on patients’ opinion of doctors [4, 11, 12, 15, 16] while studies focusing on patients’ opinions of pharmacy and pharmacy services have only been conducted through interviews and surveys [17–23]. This study will provide a novel and detailed insight into those pharmacy related issues that patients feel important to feedback to healthcare organisations.

## Aim of the study

The aim of this study is to identify the main themes contained within online patient feedback relating to patient experiences of interactions with pharmacy staff and pharmacy services.

## Ethical approval

Ethical approval was not required due to the nature of the secondary data: stories posted on Care Opinion are anonymous and publicly available data. However, this research was conducted in line with the British Psychological Society’s Ethics Guidelines for Internet-mediated Research [24]. Care Opinion is registered with the Information Commissioner’s Office (ICO) as a data controller under the Data Protection Act 2018 (https://www.careopinion.org.uk/info/privacy).

## Method

A thematic analysis of patient stories along with staff responses published was conducted between December 2018 and December 2019 on Care Opinion (https://www.careopinion.org.uk), a national online platform that allows patients from across the UK to provide an account of their healthcare experiences and for staff to respond to them. Healthcare providers (including the majority of hospitals and GP surgeries) who are subscribers will receive a notification when feedback directed at their organisation is posted by patients. They can then respond to feedback via the platform. A 6-month subscription was granted by Care Opinion for full access and the ease of data extraction, similar to a premium service paid by some NHS providers for additional functionalities (e.g. report generation, data access, and visualisation). Care Opinion interoperates with the NHS website (https://www.nhs.uk/pages/home.aspx), considered as another widely used online feedback platform where the public can rate services and leave feedback.

### Data collection

Stories relevant to pharmacy were identified from the database by applying Care Opinion search filters including the terms: pharmacy, pharmacist, pharmacy services, chemist, pharmacy staff. Patient stories were included in the study if they related to the context of pharmacy in community, hospital, or GP settings. Stories were excluded if the content only related to GP surgery, non-hospital related pharmacy services, and any non-useful descriptions where the term ‘pharmacy’ appeared in the text. Due to the lack of sensitivity of the care opinion search, two authors (JL, GG) manually screened stories for inclusion and any ambiguity was resolved by discussion with PL.

### Data analysis

To understand the content of patient feedback, Braun and Clarke’s six-phase framework [25] of thematic analysis was used to analyse the data, aided by an Microsoft Excel™ spreadsheet [26]. A data-driven, inductive approach was taken with the coding of data occurring without following any pre-existing coding framework. Initially, the extracted data were read at least once by two members of the research team so as to gain familiarity with feedback content and achieve data immersion for the development of codes. The labels for the codes derived from the initial reading were agreed upon by all researchers to form a provisional coding framework for the analysis, which were then refined through multiple iterations to produce a final coding framework. The final codes were categorised into themes and subthemes and reviewed by all researchers to ensure agreement. Counts of themes and subthemes were also recorded. The entire data was reported on three levels: community pharmacy, hospital pharmacy, and GP practice pharmacy.

A sentiment analysis of extracted patient stories was undertaken alongside the thematic analysis to add further depth and greater interpretative power through understanding patient tone within each story. This process is usually computer-assisted [27], but was done manually by two researchers (JL, GG) as the sample size made it feasible for manual annotation. Stories were read and classified as either positive, negative or mixed in relation to the pharmacy narrative.

## Results

### Thematic analysis of patient stories

Six core themes were identified from the analysis: *staff attitudes, services, errors, supply, accessibility, and system*, with each containing three sub-themes. Two hundred thirty-seven (237, 27.9%) stories were identified as containing pharmacy-related content from the initial 848 Care Opinion stories. Of the 237 stories, there were a total of 356 codes. One hundred and forty-eight (148) stories were single coded and grouped into one theme or subtheme. A further 89 stories were coded more than once and grouped into two or more themes or subthemes. Seventy-four patient stories (31.2%) were directly related to their experiences of pharmacy or of pharmacy staff, while the remaining 163 (68.8%) described patients’ overall healthcare experience with some reference to their experiences of pharmacy services or staff.

**Fig. 1:**
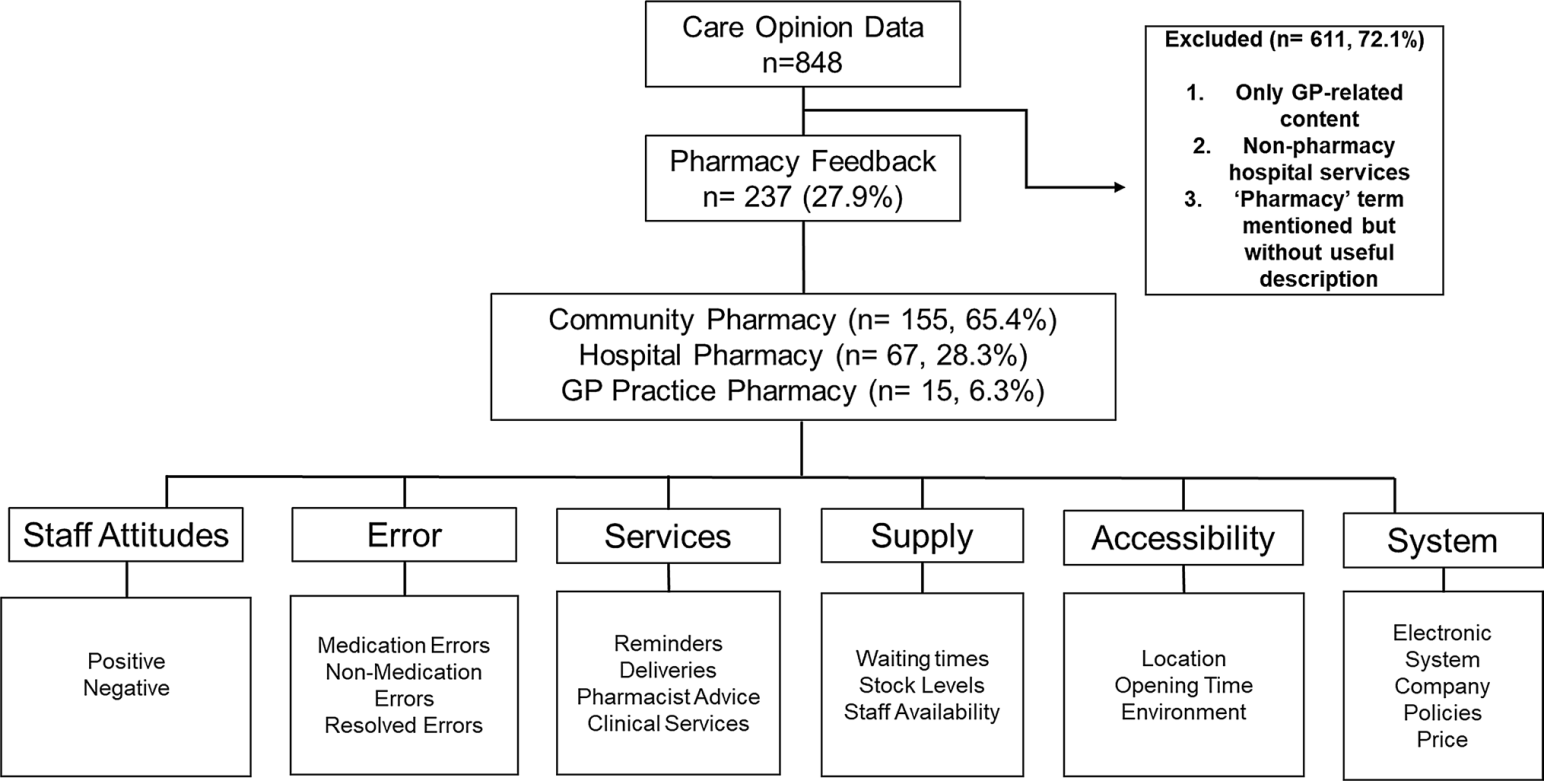
A flow diagram of the pharmacy feedback selection process, themes and subthemes

The stories were then categorised into one of three settings: community pharmacy (n = 155, 65.4%), hospital pharmacy (n = 67, 28.3%) and GP practice pharmacy (n = 15, 6.3%) for analysis. The entire process is illustrated in Fig. 1.

Overall, supply was the most common theme of pharmacy related patient stories (104, 43.9%), followed, in decreasing frequency, by the themes of staff attitudes, services, accessibility, errors and systems, each receiving 93 (39.2%), 53 (22.4%), 40 (16.9%), 29 (12.2%) and 12 (5.1%) stories respectively, as shown in Table 1. However, the lack of GP pharmacy feedback hinders the allowance of meaningful analysis for this sector.

**Table 1:** Summary of themes and subthemes contained in pharmacy related patient stories

Themes	Subthemes	Total (N = 237)	Community (N = 155)	Hospital (N = 67)	GP (N = 15)
Supply		**104 (43.9%)**	**50 (32.3%)**	**53 (79.1%)**	**1 (6.67%)**
	Waiting times	90 (38%)	40 (25.8%)	49 (73.1%)	1 (6.67%)
	Staff Availability	11 (4.64%)	4 (2.58%)	7 (10.4%)	0
	Stock levels	19 (8.02%)	12 (7.74%)	7 (10.4%)	0
Staff attitudes		**93 (39.2%)**	**65 (41.9%)**	**17 (25.4%)**	**11 (73.3%)**
	Positive	68 (28.7%)	48 (40%)	10 (14.9%)	10 (66.7%)
	Negative	26 (11%)	18 (11.7%)	7 (10.4%)	1 (6.67%)
Services		**53 (22.4%)**	**34 (21.9%)**	**12 (17.9%)**	**7 (46.7%)**
	Deliveries	5 (2.11%)	4 (2.58%)	1 (1.49%)	0
	Reminders	4 (1.69%)	2 (1.29%)	1 (1.49%)	1 (6.67%)
	Clinical Services	27 (11.4%)	16 (10.3%)	6 (8.96%)	5 (33.3%)
	*Pharmacist advice*	*21 (8.86%)*	*14 (9.03%)*	*5 (7.46%)*	*2 (13.3%)*
Accessibility		**40 (16.9%)**	**33 (22.3%)**	**7 (10.5%)**	**0**
	Location	29 (12.2%)	27 (17.4%)	2 (2.99%)	0
	Opening Times	2 (0.84%)	2 (1.29%)	0	0
	Environment	11 (4.64%)	5 (3.23%)	6 (8.96%)	0
System		**29 (12.2%)**	**21 ( 13.6%)**	**8 (11.9%)**	**0**
	Company Policies	19 (8.02%)	13 (8.39%)	6 (8.96%)	0
	Electronic System	5 (2.11%)	3 (1.94%)	2 (2.99%)	0
	Price	6 (2.53%)	6 (3.87%)	0	0
Errors		**12 (5.1%)**	**8 (5.16%)**	**4 (5.97%)**	**0**
	Medication	3 (1.27%)	3 (1.94%)	0	0
	Non-medication	7 (2.95%)	5 (3.23%)	2 (2.99%)	0
	Errors resolved	3 (1.27%)	1 (0.65%)	2 (2.99%)	0

As patient stories contained multiple subthemes their numbers do not add up to the total number of stories within a theme

### Sentiment analysis of patient stories

Among the 237 stories, a relatively equal number were classified as positive (121, 51.1%) and negative (113, 47.7%). The remaining were mixed (3, 1.3%), where both positive and negative sentiment were expressed in the narrative. However, when the data was analysed according to pharmacy sector, a majority of patients reported negatively towards hospital pharmacy (51, 76.1%). In contrast, community pharmacy had slightly more positive (86, 55.4%) than negative (68, 43.8%) feedback. In GP practices, the majority of feedback was positive (13, 86.7%). The overview of sentiment for the pharmacy themes and their respective subthemes are quantified and illustrated in Table 2.

**Table 2:** An overview of sentiment analysis for the themes and subthemes across different pharmacy sectors

Themes Subthemes	Sentiment (N = 237)	Community (N = 155)	Hospital (N = 67)	GP (N = 15)
Supply	Negative 89 (85.6%)	Negative 38 (76%)	Negative 51 (96.2%)	Positive 1 (100%)
Waiting Times	Negative 76 (84.4%)	Negative 29 (72.5%)	Negative 47 (95.9%)	Positive 1 (100%)
Stock Levels	Negative 18 (94.5%)	Negative 11 (91.7%)	Negative 7 (100%)	N/A
Staff Availability	Negative 10 (90.9%)	Negative 3 (75%)	Negative 7 (100%)	N/A
Staff Attitudes	Positive 67 (72%)	Positive 47 (72.3%)	Positive 10 (58.8%)	Positive 10 (90.9%)
Services	Positive 38 (71.7%)	Positive 24 (70.6%)	Positive 8 (66.7%)	Positive 6 (85.7%)
Deliveries	Negative 4 (80%)	Negative 3 (75%)	Positive 1 (100%)	N/A
Reminders	Positive 4 (100%)	Positive 2 (100%)	Positive 1 (100%)	Positive 1 (100%)
Clinical Services	Positive 22 (81.5%)	Positive 13 (81.3%)	Positive 4 (66.7%)	Positive 5 (100%)
Patient Advice	Positive 15 (71.4%)	Positive 10 (71.4%)	Positive 4 (80%)	N/P* 1 (50%)
Accessibility	Positive 28 (70%)	Positive 27 (81.8%)	Negative 1 (85.7%)	N/A
Location	Positive 26 (89.7%)	Positive 25 (92.6%)	N/P* 1 (50%)	N/A
Opening times	N/P* 1 (50%)	N/P* 1 (50%)	N/A	N/A
Environment	Negative 9 (81.8%)	Negative 3 (60%)	Negative 6 (100%)	N/A
System	Negative 26 (89.7%)	Negative 18 (85.7%)	Negative 8 (100%)	N/A
Company Policies	Negative 19 (100%)	Negative 13 (100%)	Negative 6 (100%)	N/A
Electronic System	Negative 4 (80%)	Negative 2 (66.7%)	Negative 2 (100%)	N/A
Price	Negative 4 (66.7%)	Negative 4 (66.7%)	N/A	N/A
Errors	Negative 10 (83.3%)	Negative 7 (87.5%)	Negative 3 (75%)	N/A

N/P* means that there is an equal distribution of negative and positive emotion. Calculation of (%) is relative to total number of stories of a theme or subtheme as seen in Table 2

### Supply

The supply of pharmacy medication constituted the largest proportion of hospital feedback (53/67, 79.1%). Patient feedback was mainly negative (89/104, 85.6%) from both community (38/50, 76%) and hospital (51/53, 96.2%) patients with waiting times being the main reason for dissatisfaction (90/104, 86.5%). Stories relating to waiting times in hospitals predominantly concerned discharge delays typically ranging from two to eight hours and outpatient medication delays; these stories made up 49/67 (73.1%) of all hospital stories. Negative feedback towards community pharmacy related to long waits (hours to days) for prescriptions. In contrast, a smaller number of positive community pharmacy stories generally praised their speed of service.

*Example (negative): ‘My experience at the pharmacy on the last four occasions have been horrendous waiting up to two hours for prescriptions to be dispensed seeing vulnerable people having to stand and wait…’ (Story 173, Hospital Pharmacy)*

Several patients described a disruption in their supply of medication, affecting the amount of time they had to wait for their prescription to be dispensed. The only positive story was an expression of surprise when a pharmacy had medication in stock.

*Example (negative): ‘...It is now Thursday night and in the unlikely event my prescription is even issued tomorrow, the pharmacy does not keep these tablets in stock, so we’re looking at next Monday, earliest.’ (Story 22, Community Pharmacy)*

### Staff attitudes

Patient feedback relating to staff attitudes was positive overall (67/93, 72%). This theme made up the highest proportion of patient feedback relating to community pharmacy (65/155, 41.9%) with most feedback being positive (47/65, 72.3%). Helpfulness, professionalism, kindness, friendliness, politeness were common terms used to describe pharmacy staff across all settings.

*Example (positive): ‘…the staff in the hospital pharmacy are some of the most patient pleasant caring people I have ever met…’ (Story 5, Community Pharmacy)*

Negative patient stories described pharmacy staff in community pharmacy and in a general practice surgery as rude, hostile and condescending. Hospital pharmacy staff were described in a small number of stories as unprofessional.

*Example (negative): ‘…Then the same pharmacy individual called my number…and from the outset seemed to find my two prescription items something highly amusing and started waving a box of laxative powders at me as if he were selling soap powder whilst at the same time almost giggling as if it were some joke…’ (Story 157, Hospital Pharmacy)*

### Services

Patient feedback was overall positive (38/53, 71.7%) regarding the services that pharmacies offer such as healthcare advice, clinical services (e.g. community pharmacy blood pressure checks, minor ailment services) as well as ordering repeat prescriptions and delivery services.

*Example (positive): ‘*The pharmacist asked me a relevant question about my symptoms. Then only after this discussion did we realise that the prescription was incorrect and hugely insufficient for my needs… It's lucky the pharmacist asked the correct question, or I would have a wholly inadequate prescription.’ (Story 175, Community Pharmacy)

Patient stories relating to Medicines Use Reviews (MURs) or the New Medicines Service (NMS) (English nationally agreed advanced services) were uncommon yet described positively:

*Example (positive): ‘I saw a doctor about my asthma who prescribed me a new inhaler. Pharmacy were very helpful and rang me 2 times to check how I was getting on with it. The 2nd call I told them I wasn’t getting on with it very well so they advised me to get another appointment.’ (Story 196, Community Pharmacy)*

Patients’ stories relating to GP pharmacy described the exceptional care they received on their medicines use and the work pharmacy staff did to ensure patients received an appropriate alternative medication in light of medication shortages.

*Example (positive): ‘…The Pharmacist was able to discuss an alternative with my GP and send a new electronic prescription to a different Pharmacy…’ (Story 120, GP Pharmacy).*

The patient story below highlighted the patient-centred care provided by a General Practice pharmacist leading to patient empowerment.

*Example (positive): ‘Following on from a change of medication required when one item was withdrawn. This was followed up by the amount of tablets I could request being dropped. Following this medication review I felt more involved in my medical care.’ (Story 183, GP Pharmacy)*

Within the hospital setting, patients described positively the service they received and the information provided by hospital pharmacists to prevent medication side effects.

*Example (positive): ‘…Pharmacist who came to the ward with prescriptions and eye drops so that one did not need to visit the pharmacy or queue, who then patiently explained to each person how the medications were to be used.’ (Story 178, Hospital Pharmacy)*

The home delivery of medication, a service offered free of charge by community pharmacies in England, was associated with a small number of negative stories (4/5, 80%). Patients complained of failed medication deliveries from their community pharmacy that caused them to wait for their medication. However, a small number of patients (2/155, 1.29%) described how they appreciated reminders via text or call to collect their medication.

### Accessibility

Patient stories often related to the accessibility of the pharmacy. Overall, accessibility was positively (28/40, 70%) described by patients. Several stories related to the convenience of community pharmacies attached to or within GP surgeries:

*Example (positive): ‘…on-site pharmacy, accessible, can always park, staff helpful and friendly and very high standard of care and expertise…’ (Story 42, Community Pharmacy)*

A minority of patient feedback related to the environment of the pharmacy (11/237, 4.64%) and these were mainly negative (9/11, 81.8%). Patients complained of crowding within some community pharmacies, whereas complaints regarding hospital pharmacy were directed at the outpatient pharmacy. Opening times of community pharmacies were appreciated in a small number of stories (2/155, 1.29%).

### Systems

This theme refers to the organisation or processes of a company including company policies, electronic systems and pricing. The theme was largely related to negative (26/29, 89.7%) patient stories. All patients describing company policies felt negatively (19/19, 100%) towards them in both hospital and community pharmacy; patients described the lack of efficiency in handling administrative and procedural problems. The most common problem experienced by patients related to the systems for handling prescriptions and communicating these processes to patients. Other procedural problems were also described e.g. complaints policy, refund policy, etc. A lack of ownership of patients’ prescription problems was an issue described in some patient stories:

*Example (negative): ‘Prescription ordering is a nightmare at this surgery for some inexplicable reason? The pharmacy blame the surgery and the surgery blame the pharmacy.’ (Story 92, Community Pharmacy)*

Complaints were also made relating to the reliability of Electronic Prescription Services (EPS), resulting in lost, as well as late, prescriptions. A lack of joined up working and information sharing between healthcare services was also evident.

*Example (negative): ‘The pharmacy could not find anything on me again a week before I am due to go away even though I spoke with the pharmacist 2 weeks ago … Funny the pharmacist couldn’t find anything to do with me yet when I had my gastroscopy yesterday they had everything on computer…’ (Story 144, Community Pharmacy)*

Price was a concern for a minority of patients who were not exempt from prescription charges and the cost of private prescriptions was occasionally mentioned as being either expensive or cheap:

*Example (negative): ‘I don't earn a lot of money so the £18 I was advised I would be refunded didn't happen and that lack of help was outrageous.’ (Story 4, Community Pharmacy)*

### Errors

There were relatively few stories relating to errors (10/237 stories, 4.22%). Errors were unsurprisingly negative experiences (10/10, 100%) for patients. There were more non-medication related errors (7/237, 2.95%) described in patient stories than medication errors (3/237, 1.27%). Patients complained of administrative errors, such as erroneous prescription charges or non-medication related advice. Medication errors were only mentioned in community pharmacy related stories whereby medications were mistakenly dispensed.

*Example (negative): ‘…Items are missing or you are given a completely different item that you did not order—which is potentially dangerous?’ (Story 92, Community Pharmacy)*

## Discussion

This is the first study to explore the content of patients’ online feedback in relation to pharmacy in the UK. The themes that we identified from patients’ stories reflected the findings of a similar study of patient feedback relating to general NHS care [16]. However, our study delved further, exploring in detail patients’ pharmacy related feedback and its variation across different pharmacy settings. Our findings provide some direction for future improvements in pharmacy by providing an insight into what patients’ value in their interactions with pharmacy services.

Our findings of feedback sentiment did not support the notion that online feedback is generally positive as sentiment was evenly split across patient stories. The most disappointment was expressed in patients’ experiences of hospital pharmacy in relation to waiting times. Overall, pharmacy waiting times were the most commonly described topic in patient stories of their pharmacy experiences. Concerns relating to hospital pharmacy waiting times corresponded with the recent NHS inpatient survey [28] that reported 41% of patients experienced a delay in discharge. This current study highlighted that patients often attribute discharge delays to long waits for medication, corroborating the inpatient survey findings that reported that 71% of patients who experienced delays perceived them to be medication-related [28]. What is not clear is whether delays in medication relate to the process of prescribing or dispensing, although studies have shown that more than half of the discharge medication processing time takes place before the prescription reaches pharmacy [29, 30]. Doctors are extremely busy dealing with competing demands and discharge prescription writing can be seen as a low priority. Pharmacist prescribers are one solution to this problem with studies demonstrating significantly improved timeliness of discharge, fewer prescribing errors and improved discharge information with the implementation of pharmacist prescribing [31, 32]. Interestingly, hospital outpatient pharmacies received many complaints of dispensing delays despite a majority of outpatient pharmacies being outsourced to private providers [33]. This finding is not captured in hospital inpatient surveys but such problems should be fed back to outpatient pharmacy teams in order to identify causes and prompt improvement.

In contrast, the nature of waiting times in community pharmacy was felt to be more complex, affected by multiple factors such as stock levels, electronic prescribing systems and involving communication between multiple organisations. Patients described poor communication between GPs and pharmacy staff as a reason for delays in obtaining prescriptions. Poor relationships and infrequent collaborative efforts between community pharmacists and GPs are common issues encountered in primary care [34]. One possible approach to improving collaboration is thought to be via GP pharmacists, who could assist with building rapport between settings, unifying and improving patient care [35]. Furthermore, stock shortages have been an increasingly encountered problem for patients due to multiple supply chain disruptions [36, 37] with a particularly difficult period arising in 2019 [37–39] from when this feedback data was extracted from CareOpinion.

However, despite the level of dissatisfaction with the supply function of pharmacy there were many positive stories in relation to staff attitudes, services, and accessibility. The professionalism of pharmacy staff, particularly in community and general practice pharmacy was highlighted in many patient stories. The UK’s Pharmacy Regulator, The General Pharmaceutical Council, sets standards for the professional behaviour of Pharmacy Professionals [40] and it was positive to see patients describe these attributes in their interactions with pharmacy staff. However, despite this, there were a small number of stories highlighting unprofessional behaviours of pharmacy staff and more could be done to ensure adherence to professional standards across all staff working within a pharmacy setting.

The services described in patients’ stories of community pharmacy lacked little explicit mention of advanced pharmacy services such as NHS England’s MURs and the NMS, this finding resonates with other studies reporting a lack of awareness and low uptake of these services [18, 21–23]. However, patients described positively their experiences of community pharmacy advice and consultation, suggesting good quality care. In hospital, the current inpatient survey revealed poor provision of medication counselling yet our patient stories did not mention this but instead expressed their appreciation towards pharmacy when counselling was given. It is possible that patients lacked familiarity with the hospital pharmacist’s role and therefore their expectations were minimal in relation to medication counselling.

Although only a small amount of GP pharmacy feedback was extracted, nearly all of it was positive, this could serve as a preliminary postulation that patients appreciate the transition of the pharmacist into more clinical and direct patient-facing roles. Nevertheless, this study highlights the need for greater public recognition of the role of pharmacists and the services they provide.

Patients appeared to appreciate the accessibility of pharmacy, highlighting opening hours and co-location in GP surgeries as positive aspects of their interactions with pharmacy. This advantage of pharmacy is commonly cited [41] and, in recent times, the accessibility of pharmacy services has been brought to the forefront of the public eye remaining patient facing throughout the Covid 19 pandemic [42].

Positively, there was little mention of errors; the majority of patient stories discussing errors provided vague details, rather than mentioning specific dispensing errors and tended to focus on administrative errors such as forgetting prescriptions. The rate of dispensing errors in the UK has been reported to be approximately 3–5% [43]. This is relatively low and not all patients who experience medication errors will experience adverse reactions [44], potentially not reporting such near misses. However, it may be that medication errors such as dispensing errors are dealt with effectively within the pharmacy without patients feeling a need to use online feedback forums as a mechanism to report such errors.

Pharmacy organisations, if not doing so already, should consider the routine use of online patient feedback in conjunction with other data to generate plans for local improvements to patient pharmacy experience. Both the CPPQ questionnaire and hospital inpatient surveys ask a small number of simplistic questions that are limited in their ability to reveal the sometimes multiple and complex issues that patients encounter. Online patient feedback, on the other hand, contains rich descriptions not constrained by predetermined topics allowing patients to provide open and honest accounts of their experiences and the issues that matter to them. Engagement of pharmacy staff in responding to pharmacy feedback may help in embracing a culture of patient-centred care, supported by a growing body of evidence that patient engagement leads to increased patient-satisfaction, positive health outcomes and efficient healthcare delivery [45]. However, there is little research regarding pharmacy professional views, understanding and use of patient feedback and such studies would be beneficial in developing recommendations for the effective use of patient feedback in practice.

### Strengths and Limitations

This study provided a rich insight into the views of patients towards pharmacy services by exploring patients’ online stories. The online nature of the patient feedback allowed for a detailed analysis of a large number of pharmacy related patient stories, an approach that, to our knowledge, is the first of its kind. However, due to the anonymity of data, it is impossible to determine patient demographics and therefore our insights might have excluded the most vulnerable patient groups as online feedback has a bias towards the young and the technology literate [11, 46]. Another limitation relates to the source of data; Care Opinion operates using a subscription approach and at present community pharmacy organisations are not generally subscribers. The majority of subscribers are NHS hospitals and General Practitioners and therefore the majority of community pharmacy feedback was sourced from stories directed towards GP practices therefore lacked detail.

### Conclusion

This study has provided a novel perspective of patients’ experiences of pharmacies in different settings. The findings reflect some known issues faced by the pharmacy sector and the wider NHS, such as the frustration caused by long waiting times, medication shortages and poor communication across healthcare organisations. It also highlighted the benefits of the pharmacy sector such as its accessibility, clinical services and the professionalism of pharmacy staff. Of novel insight was the appreciation of clinical services, particularly those provided by GP pharmacists. Further research is required to understand how online patient feedback can be used effectively to improve pharmacy healthcare practices and policy and ultimately improve the care patients receive.

## References

[1] Department of Health (1997). The New NHS: Modern, Dependable Cm 3807.

[2] DeCourcy A, West E, Barron D (2012). The national adult inpatient survey conducted in the english national health service from 2002 to 2009: how have the data been used and what do we know as a result?. BMC Health Serv Res.

[3] Department of Health (2010). Equity and excellence: liberating the NHS Cm 7881.

[4] Patel S, Cain R, Neailey K, Hooberman L (2015). General practitioners’ concerns about online patient feedback: findings from a descriptive exploratory qualitative study in England. J Med Internet Res.

[5] van Velthoven MH, Atherton H, Powell J (2018). A cross sectional survey of the UK public to understand use of online ratings and reviews of health services. Patient Educ Couns.

[6] Burkle CM, Keegan MT (2015). Popularity of internet physician rating sites and their apparent influence on patients' choices of physicians. BMC Health Serv Res.

[7] Galizzi MM, Miraldo M, Stavropoulou C, Desai M, Jayatunga W, Joshi M (2012). Who is more likely to use doctor-rating websites, and why? A cross-sectional study in London. BMJ Open.

[8] NHS. The Long Term Plan [Internet]. England: NHS; 2019 Jan 7 [updated 2019 Aug 21;cited 2020 Jul 15]. https://www.longtermplan.nhs.uk/

[9] Atherton H, Fleming J, Williams V, Powell J (2019). Online patient feedback: a cross-sectional survey of the attitudes and experiences of United Kingdom health care professionals. J Health Serv Res Policy.

[10] Gao GG, McCullough JS, Agarwal R, Jha AK (2012). A changing landscape of physician quality reporting: analysis of patients' online ratings of their physicians over a 5-year period. J Med Internet Res.

[11] Powell J, Atherton H, Williams V, Mazanderani F, Dudhwala F, Woolgar S (2019). Using online patient feedback to improve NHS services: the INQUIRE multimethod study. Health Serv Deliv Res.

[12] Boylan AM, Williams V, Powell J (2020). Online patient feedback: a scoping review and stakeholder consultation to guide health policy. J Health Serv Res Policy.

[13] Coulter A, Locock L, Ziebland S, Calabrese J (2014). Collecting data on patient experience is not enough: they must be used to improve care. BMJ.

[14] Ramsey LP, Sheard L, Lawton R, O’Hara J (2019). How do healthcare staff respond to patient experience feedback online? A typology of responses published on care opinion. Patient Exp J.

[15] Boylan A-M, Turk A, van Velthoven MH, Powell J (2020). Online patient feedback as a measure of quality in primary care: a multimethod study using correlation and qualitative analysis. BMJ Open.

[16] Brookes G, Baker P (2017). What does patient feedback reveal about the NHS? A mixed methods study of comments posted to the NHS Choices online service. BMJ Open..

[17] Keshishian F, Colodny N, Boone RT (2008). Physician-patient and pharmacist-patient communication: geriatrics' perceptions and opinions. Patient Educ Couns.

[18] Hindi AMK, Schafheutle EI, Jacobs S (2018). Patient and public perspectives of community pharmacies in the United Kingdom: a systematic review. Health Expect.

[19] Morecroft CW, Thornton D, Caldwell NA (2015). Inpatients' expectations and experiences of hospital pharmacy services: qualitative study. Health Expect.

[20] Hobson RJ, Scott J, Sutton J (2010). Pharmacists and nurses as independent prescribers: exploring the patient's perspective. Fam Pract.

[21] Rodgers RM, Gammie SM, Loo RL, Corlett SA, Krska J (2016). Comparison of pharmacist and public views and experiences of community pharmacy medicines-related services in England. Patient Prefer Adherence.

[22] Merks P, ŚWieczkowski D, Jaguszewski MJ (2016). Patients' perception of pharmaceutical services available in a community pharmacy among patients living in a rural area of the United Kingdom. Pharm Pract (Granada).

[23] King PK, Martin SJ, Betka EM (2017). Patient awareness and expectations of pharmacist services during hospital stay. J Pharm Pract.

[24] British Psychological Society. Ethics Guidelines for Internet-mediated Research NF206/04.2017 [Internet]. Leicester: Hewson C, Buchanan T, Brown I, Coulson N, Hagger-Johnson G, Joinson A, Krotoski A, Oates J.; 2017 [cited 2021 May 14]. https://www.bps.org.uk/sites/bps.org.uk/files/Policy/Policy%20-%20Files/Ethics%20Guidelines%20for%20Internet-mediated%20Research%20(2017).pdf

[25] Braun V, Clarke V (2006). Using thematic analysis in psychology. Qual Res Psychol.

[26] Bree R, Gallagher G.: Using microsoft excel to code and thematically analyse qualitative data: a simple, cost-effective approach. AISHE-J - All Irel J Teach Learn High Educ. 2016; 8(2)

[27] D’Andrea A, Ferri F, Grifoni P, Guzzo T (2015). Approaches, tools and applications for sentiment analysis implementation. Int J Comput Appl.

[28] Care Quality Commission (CQC). 2019 Adult Inpatient Survey: Statistical Release. England: Care Quality Commission; 2019 July [updated 2020 July 02, cited 2021 May 14] https://www.cqc.org.uk/sites/default/files/20200702_ip19_statisticalrelease.pdf

[29] Marvin V, Kuo S, Linnard D (2013). DSL-007|does pharmacy contribute to delays in hospital discharge?. Eur J Hosp Pharm Sci Practice.

[30] Green CF, Hunter L, Jones L, Morris K (2015). The TTO journey: how much of it is actually in pharmacy?. Pharm Manag.

[31] Physick A, Smolski K, Mann S, Price G (2016). Pharmacy innovation at discharge - impact of pharmacist non-medical prescribing on quality and streamlining processes. J Med Optim.

[32] NHS Improvement. Rapid improvement Guide to: optimising medicines discharge to improve patient flow [Internet]. [cited 2021 May 14]. https://www.england.nhs.uk/south/wp-content/uploads/sites/6/2016/12/rig-optimising-medicines-discharge.pdf

[33] NHS. Outsourcing Hospital Pharmacy Outpatient Dispensing (OPD) Services – Challenges and Opportunities [Internet]. England: Pat R, Anderson M. 2012 June 10 [cited 2021 May 14] https://www.sps.nhs.uk/articles/outsourcing-hospital-pharmacy-outpatient-dispensing-opd-services-challenges-and-opportunities/

[34] Hindi AMK, Jacobs S, Schafheutle EI (2019). Solidarity or dissonance? A systematic review of pharmacist and GP views on community pharmacy services in the UK. Health Soc Care Commun.

[35] Karampatakis GD, Patel N, Stretch G (2020). Community pharmacy teams’ experiences of general practice-based pharmacists: an exploratory qualitative study. BMC Health Serv Res.

[36] National Health Service (NHS). MCG advice on manging medicines stock shortages in primary care [Internet]. Surrey: NHS; 2020[cited 2021 May 14]. https://psnc.org.uk/communitypharmacyss/wp-content/uploads/sites/121/2018/09/MCG-advice-on-managing-medicines-stock-shortages-Apr-2018-final-1.pdf

[37] European Association of Hospital Pharmacists (EAHP). 2019 EAHP Medicines Shortages Report [Internet]. Brussels: European Association of Hospital Pharmacists; 2020 [cited 2021 May 14] https://www.eahp.eu/sites/default/files/eahp_2019_medicines_shortages_report.pdf

[38] European Association of Hospital Pharmacists (EAHP). 2018 EAHP Medicines Shortages Report [Internet]. Brussels: European Association of Hospital Pharmacists; 2018 Nov[ cited 2021 May 14] https://www.eahp.eu/sites/default/files/report_medicines_shortages2018.pdf

[39] Pharmaceutical Group of European Union (PGEU). Press Release PGEU Medicine Shortages Survey Results [Internet]. Brussels: Pharmaceutical Group of European Union (PGEU); 2019[cited 2021 May 2021] https://www.pgeu.eu/wp-content/uploads/2020/01/PR-PGEU-publishes-results-of-Medicine-Shortages-Survey-2019-2.pdf

[40] General Pharmaceutical Council. Standards for pharmacy professionals May 2017 [Internet]. Great Britain: General Pharmaceutical Council. 2017 May [cited 2021 May 14] https://www.pharmacyregulation.org/sites/default/files/standards_for_pharmacy_professionals_may_2017_0.pdf.

[41] Jesson JK, Wilson KA (2003). One-stop health centres: what co-location means for pharmacy. Health Place.

[42] All-Party Pharmacy Group. All-Party Pharmacy Group Flash Inquiry. The impact of the COVID-19 pandemic on pharmacy and pharmacy teams [Internet]. England: All-Party Pharmacy Group. 2020 [cited 2021 May 14] https://static1.squarespace.com/static/5d91e828ed9a60047a7bd8f0/t/5fd70d0bdf2f0204c24da919/1607929102553/APPG_Flash_Inquiry.pdf

[43] Ashcroft DM, Quinlan P, Blenkinsopp A (2005). Prospective study of the incidence, nature and causes of dispensing errors in community pharmacies. Pharmacoepidemiol Drug Saf.

[44] Cheong V-L, Tomlinson J, Khan S, Petty D (2019). Medicines-related harm in the elderly post-hospital discharge. Prescriber.

[45] Sheard L, Peacock R, Marsh C, Lawton R (2019). What's the problem with patient experience feedback? A macro and micro understanding, based on findings from a three-site UK qualitative study. Health Expect.

[46] Rozenblum R, Miller P, Pearson D, Marelli A, Grando M, Rozenblum R, Bates D (2015). Patient-centered healthcare, patient engagement and health information technology : the perfect storm. Information technology for patient empowerment in healthcare.

